# Training Deep Spiking Neural Networks Using Backpropagation

**DOI:** 10.3389/fnins.2016.00508

**Published:** 2016-11-08

**Authors:** Jun Haeng Lee, Tobi Delbruck, Michael Pfeiffer

**Affiliations:** ^1^Samsung Advanced Institute of Technology, Samsung ElectronicsSuwon, South Korea; ^2^Institute of Neuroinformatics, University of Zurich and ETH ZurichZurich, Switzerland

**Keywords:** spiking neural network, deep neural network, backpropagation, neuromorphic, DVS, MNIST, N-MNIST

## Abstract

Deep spiking neural networks (SNNs) hold the potential for improving the latency and energy efficiency of deep neural networks through data-driven event-based computation. However, training such networks is difficult due to the non-differentiable nature of spike events. In this paper, we introduce a novel technique, which treats the membrane potentials of spiking neurons as differentiable signals, where discontinuities at spike times are considered as noise. This enables an error backpropagation mechanism for deep SNNs that follows the same principles as in conventional deep networks, but works directly on spike signals and membrane potentials. Compared with previous methods relying on indirect training and conversion, our technique has the potential to capture the statistics of spikes more precisely. We evaluate the proposed framework on artificially generated events from the original MNIST handwritten digit benchmark, and also on the N-MNIST benchmark recorded with an event-based dynamic vision sensor, in which the proposed method reduces the error rate by a factor of more than three compared to the best previous SNN, and also achieves a higher accuracy than a conventional convolutional neural network (CNN) trained and tested on the same data. We demonstrate in the context of the MNIST task that thanks to their event-driven operation, deep SNNs (both fully connected and convolutional) trained with our method achieve accuracy equivalent with conventional neural networks. In the N-MNIST example, equivalent accuracy is achieved with about five times fewer computational operations.

## 1. Introduction

Deep learning is achieving outstanding results in various machine learning tasks (He et al., 2015a; LeCun et al., 2015), but for applications that require real-time interaction with the real environment, the repeated and often redundant update of large numbers of units becomes a bottleneck for efficiency. An alternative has been proposed in the form of spiking neural networks (SNNs), a major research topic in theoretical neuroscience and neuromorphic engineering. SNNs exploit event-based, data-driven updates to gain efficiency, especially if they are combined with inputs from event-based sensors, which reduce redundant information based on asynchronous event processing (Camunas-Mesa et al., 2012; O'Connor et al., 2013; Merolla et al., 2014; Neil and Liu, 2016). This feature makes spiking systems attractive for real-time applications where speed and power consumption are important factors, especially once adequate neuromorphic hardware platforms become more widely available. Even though in theory (Maass and Markram, 2004) SNNs have been shown to be as computationally powerful as conventional artificial neural networks (ANNs; this term will be used to describe conventional deep neural networks in contrast with SNNs), practically SNNs have not quite reached the same accuracy levels of ANNs in traditional machine learning tasks. A major reason for this is the lack of adequate training algorithms for deep SNNs, since spike signals (i.e., discrete events produced by a spiking neuron whenever its internal state crosses a threshold condition) are not differentiable, but differentiable activation functions are fundamental for using error backpropagation, which is still by far the most widely used algorithm for training deep neural networks.

A recently proposed solution is to use different data representations between training and processing, i.e., training a conventional ANN and developing conversion algorithms that transfer the weights into equivalent deep SNNs (O'Connor et al., 2013; Diehl et al., 2015; Esser et al., 2015; Hunsberger and Eliasmith, 2015). However, in these methods, details of statistics in spike trains that go beyond ideal mean rate modeling, such as required for processing practical event-based sensor data cannot be precisely represented by the signals used for training. It is therefore desirable to devise learning rules operating directly on spike trains, but so far it has only been possible to train single layers, and use unsupervised learning rules, which leads to a deterioration of accuracy (Masquelier and Thorpe, 2007; Neftci et al., 2014; Diehl and Cook, 2015). An alternative approach has recently been introduced by O'Connor and Welling (2016), in which a SNN learns from spikes, but requires keeping statistics for computing stochastic gradient descent (SGD) updates in order to approximate a conventional ANN.

In this paper we introduce a novel supervised learning method for SNNs, which closely follows the successful backpropagation algorithm for deep ANNs, but here is used to train general forms of deep SNNs directly from spike signals. This framework includes both fully connected and convolutional SNNs, SNNs with leaky membrane potential, and layers implementing spiking winner-takes-all (WTA) circuits. The key idea of our approach is to generate a continuous and differentiable signal on which SGD can work, using low-pass filtered spiking signals added onto the membrane potential and treating abrupt changes of the membrane potential as noise during error backpropagation. Additional techniques are presented that address particular challenges of SNN training: Spiking neurons typically require large thresholds to achieve stability and reasonable firing rates, but large thresholds may result in many “dead” neurons, which do not participate in the optimization during training. Novel regularization and normalization techniques are proposed that contribute to stable and balanced learning. Our techniques lay the foundations for closing the performance gap between SNNs and ANNs, and promote their use for practical applications.

### 1.1. Related work

Gradient descent methods for SNNs have not been deeply investigated because both spike trains and the underlying membrane potentials are not differentiable at the time of spikes. The most successful approaches to date have used indirect methods, such as training a network in the continuous rate domain and converting it into a spiking version. O'Connor et al. (2013) pioneered this area by training a spiking deep belief network based on the Siegert event-rate approximation model. However, on the MNIST hand written digit classification task (LeCun et al., 1998), which is nowadays almost perfectly solved by ANNs (0.21% error rate in Wan et al., 2013), their approach only reached an accuracy around 94.09%. Hunsberger and Eliasmith (2015) used the softened rate model, in which a hard threshold in the response function of leaky integrate and fire (LIF) neuron is replaced with a continuous differentiable function to make it amenable to use in backpropagation. After training an ANN with the rate model they converted it into a SNN consisting of LIF neurons. With the help of pre-training based on denoising autoencoders they achieved 98.6% in the permutation-invariant (PI) MNIST task (see Section 3.1). Diehl et al. (2015) trained deep neural networks with conventional deep learning techniques and additional constraints necessary for conversion to SNNs. After training, the ANN units were converted into non-leaky spiking neurons and the performance was optimized by normalizing weight parameters. This approach resulted in the current state-of-the-art accuracy for SNNs of 98.64% in the PI MNIST task. Esser et al. (2015) used a differentiable probabilistic spiking neuron model for training and statistically sampled the trained network for deployment. In all of these methods, training was performed indirectly using continuous signals, which may not capture important statistics of spikes generated by real sensors used during processing. Even though SNNs are well-suited for processing signals from event-based sensors such as the Dynamic Vision Sensor (DVS) (Lichtsteiner et al., 2008), the previous SNN training models require removing time information and generating image frames from the event streams. Instead, in this article we use the same signal format for training and processing deep SNNs, and can thus train SNNs directly on spatio-temporal event streams considering non-ideal factors such as pixel variation in sensors. This is demonstrated on the neuromorphic N-MNIST benchmark dataset (Orchard et al., 2015), achieving higher accuracy with a smaller number of neurons than all previous attempts that ignored spike timing by using event-rate approximation models for training.

## 2. Materials and methods

### 2.1. Spiking neural networks

In this article we study two types of networks: Fully connected SNNs with multiple hidden layers and convolutional SNNs. Let *M* and *N* be the number of synapses of a neuron and the number of neurons in a layer, respectively. On the other hand, *m* and *n* are the number of active synapses (i.e., synapses receiving spike inputs) of a neuron and the number of active neurons (sending spike outputs) in a layer during the presentation of an input sample. We will also use the simplified form of indices for active synapses and neurons throughout the paper as

$$\begin{array}{l} Activesynapses:\;\{v_{1},\cdots ,v_{m}\}\to \{1,\cdots ,m\}\\ Activeneurons:\;\{u_{1},\cdots ,u_{n}\}\to \{1,\cdots ,n\} \end{array}$$

Thus, if an index *i*, *j*, or *k* is used for a synapse over [1, *m*] or a neuron over [1, *n*] (e.g., in Equation 5), then it actually represents an index of an active synapse (*v_i_*) or an active neuron (*u_j_*).

#### 2.1.1. Leaky integrate-and-fire (LIF) neuron

The LIF neuron is one of the simplest models used for describing dynamics of spiking neurons (Gerstner and Kistler, 2002). Since the states of LIF neurons can be updated asynchronously based solely on the timing of input events (i.e., without timestepped integration), LIF is computationally efficient. For a given input spike the membrane potential of a LIF neuron can be updated as

(1) $$\begin{array}{l} V_{mp}\left(t_{p}\right)=V_{mp}\left(t_{p-1}\right)e^{\frac{t_{p-1}-t_{p}}{\tau_{mp}}}+w_{i}^{\left(p\right)}w_{dyn}, \end{array}$$

where *V*_*mp*_ is the membrane potential, τ_*mp*_ is the membrane time constant, *t*_*p*_ and *t*_*p*−1_ are the present and previous input spike times, $w_{i}^{\left(p\right)}$ is the synaptic weight of the *i*-th synapse (through which the present *p*-th input spike arrives). We introduce here a dynamic weight *w*_*dyn*_, which controls the refractory period following

(2) $$w_{dyn}=\{\begin{array}{ll} {\left(\Delta_{t}/T_{ref}\right)}^{2} & \text{ if }\Delta_{t}<T_{ref}\text{ and }w_{dyn}<1\\ 1 & \text{ otherwise} \end{array}$$

where *T*_*ref*_ is the maximum duration of the refractory period, and Δ_*t*_ = *t*_out_ − *t*_*p*_, where *t*_out_ is the time of the latest output spike produced by the neuron or an external trigger signal through lateral inhibition as discussed in Section 2.1.2. Thus, the effect of input spikes on *V*_*mp*_ is suppressed for a short period of time *T*_*ref*_ after an output spike. *w*_*dyn*_ recovers quadratically to 1 after the output spike at *t*_out_. Since *w*_*dyn*_ is a neuron parameter and applied to all synapses identically, it is different from short-term plasticity, which is a synapse specific mechanism. The motivation to use dynamic weights instead of simpler refractory mechanisms, such as simply blocking the generation of output spikes, is that it allows controlling refractory states by external mechanisms. One example is the introduction of WTA circuits in Section 2.1.2, where lateral inhibition simultaneously puts all neurons competing in a WTA into the refractory state. This ensures that the winning neuron gets another chance to win the competition, since otherwise another neuron could fire while only the winner has to reset its membrane potential after generating a spike.

When *V*_*mp*_ crosses the threshold value *V*_*th*_, the LIF neuron generates an output spike and *V*_*mp*_ is decreased by the amount of the threshold:

(3) $$\begin{array}{l} V_{mp}\left(t_{p}^{+}\right)=V_{mp}\left(t_{p}\right)-V_{th}, \end{array}$$

where $t_{p}^{+}$ is the time right after the reset. A lower bound for the membrane potential is set at −*V*_*th*_, and *V*_*mp*_ is clipped whenever it falls below this value. This strategy helps balancing the participation of neurons during training by preventing neurons from having highly negative membrane potentials. We will revisit this issue when we introduce threshold regularization in Section 2.3.2.

#### 2.1.2. Winner-take-all (WTA) circuit

We found that the accuracy of SNNs could be improved by introducing a competitive recurrent architecture in the form of adding WTA circuits in certain layers. In a WTA circuit, multiple neurons form a group with lateral inhibitory connections. Thus, as soon as any neuron produces an output spike, it inhibits all other neurons in the circuit and prevents them from spiking (Rozell et al., 2008; Oster et al., 2009). In this work, all lateral connections in a WTA circuit have the same strength, which reduces memory and computational costs for implementing them. The amount of lateral inhibition applied to the membrane potential is proportional to the inhibited neuron's membrane potential threshold (the exact form is defined in Equation 5 in Section 2.2.2). With this scheme, lateral connections inhibit neurons having small *V*_*th*_ weakly and those having large *V*_*th*_ strongly. This improves the balance of activities among neurons during training since neurons with higher activities have larger *V*_*th*_ due to the threshold regularization scheme described in Section 2.3.2. Furthermore, as described previously in Section 2.1.1, lateral inhibition is used to put the dynamic weights of all inhibited neurons in a WTA circuit into the refractory state. As shown in **Figure 3** and discussed later in Section 3.1, we found that adding WTA circuits both improves classification accuracy, and improves the stability and speed of convergence during training.

### 2.2. Using backpropagation in SNNs

In order to derive and apply the backpropagation equations for training SNNs, after summarizing the classical backpropagation method (Rumelhart and Zipser, 1985) we derive differentiable transfer functions for spiking neurons in WTA configuration. Furthermore, we introduce simple methods to initialize parameters and normalize backpropagating errors to address vanishing or exploding gradients, and to stabilize training. These are variations of successful methods used commonly in deep learning, but adapted to the specific requirements of SNNs.

#### 2.2.1. Backpropagation revisited

Neural networks are typically optimized by SGD, meaning that the vector of network parameters or weights θ is moved in the direction of the negative gradient of some loss function *L* according to θ = θ − η*∂L*/∂θ, where η is the learning rate. The backpropagation algorithm uses the chain rule to compute the partial derivatives ∂*L*/∂θ. For completeness we provide here a summary of backprop for conventional fully-connected deep neural networks:

1. Propagation inputs in the forward direction to compute the pre-activations (*z*^(*l*)^) and activations (*a*^(*l*)^ = *f*^(*l*)^(*z*^(*l*)^)) for all the layers up to the output layer *l*_*n*_*l*__, where *f* is the transfer function of units.
2. Calculate the error at the output layer:
  $$\delta^{\left(n_{l}\right)}=\frac{\partial L(a^{\left(n_{l}\right)},y)}{\partial z^{\left(n_{l}\right)}}=\frac{\partial L(a^{\left(n_{l}\right)},y)}{\partial a^{\left(n_{l}\right)}}\cdot f^{\prime }(z^{\left(n_{l}\right)})$$
  where y is the label vector indicating the desired output activation and · is element-wise multiplication.
3. Backpropagate the error to lower layers *l* = *n*_*l*_ − 1, *n*_*l*_ − 2, …, 2:
  $$\delta^{\left(l\right)}=\left({\left(W^{\left(l\right)}\right)}^{T}\delta^{\left(l\;+\;1\right)}\right)\cdot f^{\prime }(z^{\left(l\right)})$$
  where *W*^(*l*)^ is the weight matrix of the layer *l*.
4. Compute the partial derivatives for the update:
  $$\begin{array}{l} \nabla_{W^{\left(l\right)}}L=\delta^{\left(l\;+\;1\right)}{\left(a^{\left(l\right)}\right)}^{T}\\ \nabla_{b^{\left(l\right)}}L=\delta^{\left(l\;+\;1\right)} \end{array}$$
  where *b*^(*l*)^ is the bias vector of the layer *l*.
5. Update the parameters:
  $$\begin{array}{l} W^{\left(l\right)}=W^{\left(l\right)}-\eta \nabla_{W^{\left(l\right)}}L\\ b^{\left(l\right)}=b^{\left(l\right)}-\eta \nabla_{b^{\left(l\right)}}L \end{array}$$

#### 2.2.2. Transfer function and derivatives

Starting from the event-based update of the membrane potentials in Equation (1), we can define the accumulated effect (normalized by synaptic weight) of the *k*-th active input synapse onto the membrane potential of a target neuron as *x*_*k*_(*t*). Similarly, the generation of spikes in neuron *i* acts on its own membrane potential via the term *a*_*i*_, which is due to the reset in Equation (3) (normalized by *V*_*th*_). Both *x*_*k*_ and *a*_*i*_ can be expressed as sums of exponentially decaying terms

(4) $$\begin{array}{l} x_{k}\left(t\right)=\underset{p}{\sum }\exp\left(\frac{t_{p}-t}{\tau_{\text{mp}}}\right),\;a_{i}\left(t\right)=\underset{q}{\sum }\exp\left(\frac{t_{q}-t}{\tau_{\text{mp}}}\right), \end{array}$$

where the first sum is over all input spike times *t*_*p*_ < *t* at the *k*-th input synapse, and the second sum is over the output spike times *t*_*q*_ < *t* for *a*_*i*_. The accumulated effects of lateral inhibitory signals in WTA circuits can be expressed analogously to Equation (4). The activities in Equation (4) are real-valued and continuous except for the time points where spikes occur and the activities jump up. We use these numerically computed lowpass-filtered activities for backpropagation instead of directly using spike signals.

Ignoring the effect of refractory periods for now, the membrane potential of the *i*-th active neuron in a WTA circuit can be written in terms of *x*_*k*_ and *a*_*i*_ defined in Equation (4) as

(5) $$\begin{array}{l} V_{mp,i}\left(t\right)=\overset{m}{\underset{k=1}{\sum }}w_{ik}x_{k}\left(t\right)-V_{th,i}a_{i}\left(t\right)+\sigma V_{th,i}\overset{n}{\underset{j=1,j\neq i}{\sum }}\kappa_{ij}a_{j}\left(t\right). \end{array}$$

**Figure 1:**
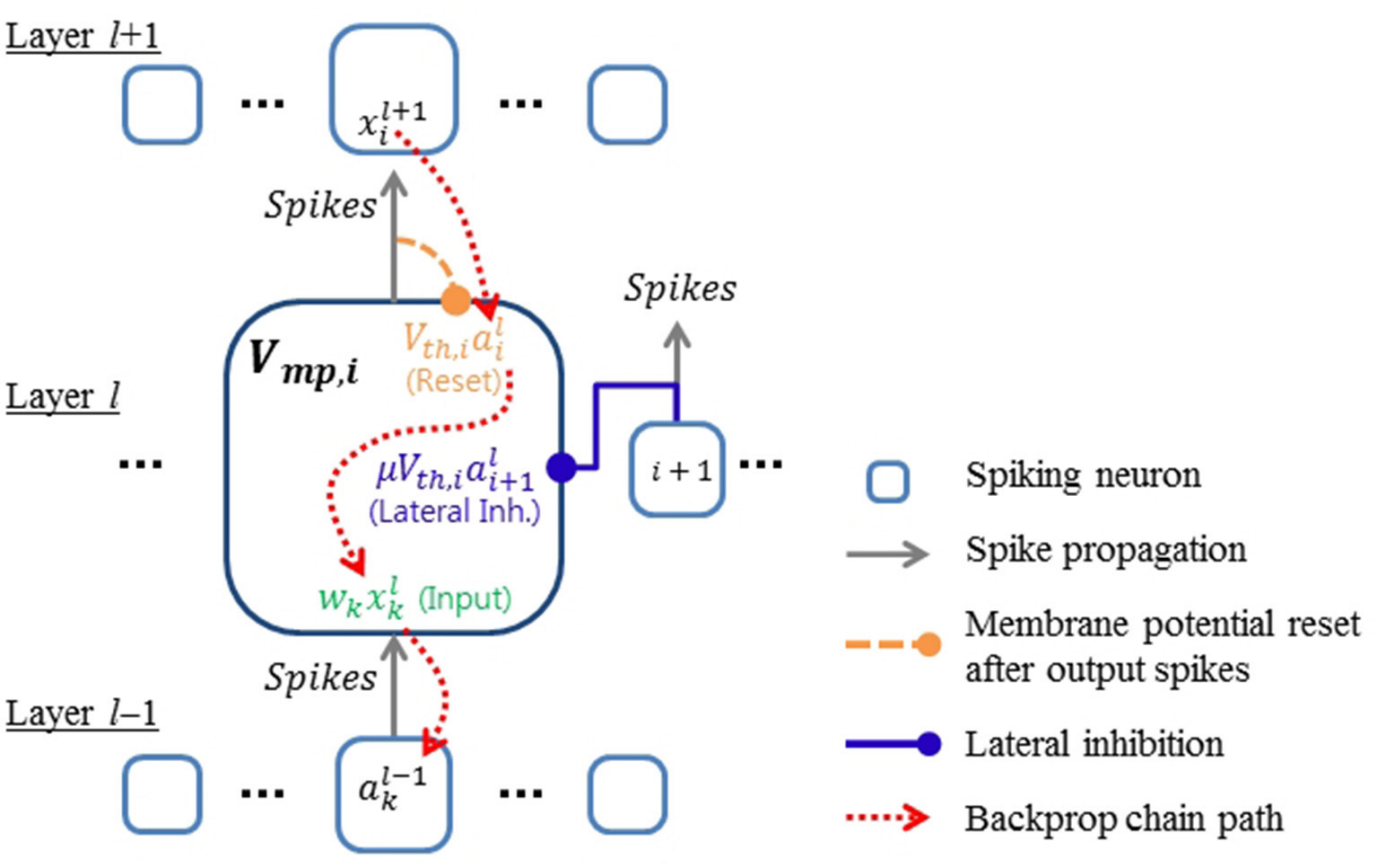
**Conceptual diagram showing the relationship between signals in the proposed spiking neural network model**. Error gradients are back-propagated through the components of the membrane potential defined in Equation (4).

The terms on the right side represent the input, membrane potential resets, and lateral inhibition, respectively. κ_*ij*_ is the strength of lateral inhibition (−1 ≤ κ_*ij*_ ≤ 0) from the *j*-th active neuron to the *i*-th active neuron, and σ is the expected efficacy of lateral inhibition. σ should be smaller than 1, since lateral inhibitions can affect the membrane potential only down to its lower bound (i.e., −*V*_*th*_). We found a value of σ ≈ 0.5 to work well in practice. Equation (5) reveals the relationship between inputs and outputs of spiking neurons which is not clearly shown in Equations (1) and (3). Nonlinear activation of neurons is considered in Equation (5) by including only active synapses and neurons. Figure 1 shows the relationship between signals presented in Equations (4) and (5). Since the output (*a*_*i*_) of the current layer becomes the input (*x*_*k*_) of the next layer if all the neurons have same τ_*mp*_, Equation (5) provides the basis for deriving the backpropagation algorithm via the chain rule.

Differentiation is not defined in Equation (4) at the moment of each spike because there is a discontinuous step jump. However, we propose here to ignore these fluctuations, and treat Equations (4) and (5) as if they were differentiable continuous signals to derive the necessary error gradients for backpropagation. In previous works (O'Connor et al., 2013; Diehl et al., 2015; Esser et al., 2015; Hunsberger and Eliasmith, 2015), continuous variables were introduced as a surrogate for *x*_*k*_ and *a*_*i*_ in Equation (5) for backpropagation. In this work, however, we directly use the contribution of spike signals to the membrane potential as defined in Equation (4). Thus, the real statistics of spike signals, including temporal effects such as synchrony between inputs, can influence the training process. Ignoring the step jumps caused by spikes in the calculation of gradients might of course introduce errors, but as our results show, in practice this seems to have very little influence on SNN training. A potential explanation for this robustness of our training scheme is that by treating the signals in Equation (4) as continuous signals that fluctuate suddenly at times of spikes, we achieve a similar positive effect as the widely used approach of noise injection during training, which can improve the generalization capability of neural networks (Vincent et al., 2008). In the case of SNNs, several papers have used the trick of treating spike-induced abrupt changes as noise for gradient descent optimization (Bengio et al., 2015; Hunsberger and Eliasmith, 2015). However, in these cases the model added Gaussian random noise instead of spike-induced perturbations. In this work, we directly use the actual contribution of spike signals to the membrane potential as described in Equation (4) for training SNNs. Our results show empirically that this approach works well for learning in SNNs where information is encoded in spike rates. Importantly, the presented framework also provides the basis for utilizing specific spatio-temporal codes, which we demonstrate on a task using inputs from event-based sensors.

For the backpropagation equations it is necessary to obtain the transfer functions of LIF neurons in WTA circuits (which generalizes to non-WTA layers by setting κ_*ij*_ = 0 for all *i* and *j*). For this we set the residual *V*_*mp*_ term in the left side of Equation (5) to zero (since it is not relevant to the transfer function), resulting in the transfer function

(6) $$\begin{array}{l} a_{i}\approx \frac{s_{i}}{V_{th,i}}+\sigma \overset{n}{\underset{j=1,j\neq i}{\sum }}\kappa_{ij}a_{j},\text{where}s_{i}=\overset{m}{\underset{k=1}{\sum }}w_{ik}x_{k}. \end{array}$$

Refractory periods are not considered here since the activity of neurons in SNNs is rarely dominated by refractory periods in a normal operating regime. For example, we used a refractory period of 1 ms and the event rates of individual neurons were kept within a few tens of events per second (eps). Equation (6) is consistent with (4.9) in Gerstner and Kistler (2002) without WTA terms. The equation can also be simplified to a spiking version of a rectified-linear unit by introducing a unit threshold and non-leaky membrane potential as in O'Connor and Welling (2016).

Directly differentiating Equation (6) yields the backpropagation equations

(7) $$\begin{array}{rcl} \frac{\partial a_{i}}{\partial s_{i}} & \approx & \frac{1}{V_{th,i}},\;\frac{\partial a_{i}}{\partial w_{ik}}\approx \frac{\partial a_{i}}{\partial s_{i}}x_{k},\\ \frac{\partial a_{i}}{\partial V_{th,i}} & \approx & \frac{\partial a_{i}}{\partial s_{i}}\left(-a_{i}+\sigma \overset{n}{\underset{j\neq i}{\sum }}\kappa_{ij}a_{j}\right),\\ \frac{\partial a_{i}}{\partial \kappa_{ih}} & \approx & \frac{\partial a_{i}}{\partial s_{i}}\left(\sigma V_{th,i}a_{h}\right), \end{array}$$

(8) $$\left[\begin{array}{c} \frac{\partial a_{1}}{\partial x_{k}}\\ \vdots \\ \frac{\partial a_{1}}{\partial x_{k}} \end{array}\right]\approx \frac{1}{\sigma }{\left[\begin{array}{ccc} q & \cdots & -\kappa_{1n}\\ \vdots & \ddots & \vdots \\ -\kappa_{n1} & \cdots & q \end{array}\right]}^{-1}\left[\begin{array}{c} \frac{w_{1k}}{V_{th,1}}\\ \vdots \\ \frac{w_{nk}}{V_{th,n}} \end{array}\right]$$

where *q* = 1/σ. When all the lateral inhibitory connections have the same strength (κ_*ij*_ = μ, ∀*i, j*) and are not learned, ∂*a*_*i*_/∂κ_*ih*_ is not necessary and Equation (8) can be simplified to

(9) $$\begin{array}{l} \frac{\partial a_{i}}{\partial x_{k}}\approx \frac{\partial a_{i}}{\partial s_{i}}\frac{1}{\left(1-\mu \sigma \right)}\left(w_{ik}-\frac{\mu \sigma V_{th,i}}{1+\mu \sigma \left(n-1\right)}\overset{n}{\underset{j=1}{\sum }}\frac{w_{jk}}{V_{th,j}}\right). \end{array}$$

By inserting the above derivatives in Equations (7) and (9) into the standard error backpropagation algorithm, we obtain an effective learning rule for SNNs. We consider only the first-order effect of the lateral connections in the derivation of gradients. Higher-order terms propagating back through multiple lateral connections are neglected for simplicity. This is mainly because all the lateral connections considered here are inhibitory. For inhibitory lateral connections, the effect of small parameter changes decays rapidly with connection distance. Thus, first-order approximation saves a lot of computational cost without loss of accuracy.

#### 2.2.3. Weight initialization and backprop error normalization

Good initialization of weight parameters in supervised learning is critical to handle the exploding or vanishing gradients problem in deep neural networks (Glorot and Bengio, 2010; He et al., 2015b). The basic idea behind those methods is to maintain the balance of forward activations and backward propagating errors among layers. Recently, the batch normalization technique has been proposed to make sure that such balance is maintained through the whole training process (Ioffe and Szegedy, 2015). However, normalization of activities as in the batch normalization scheme is difficult for SNNs, because there is no efficient method for amplifying event rates above the input rate. The initialization methods proposed in Glorot and Bengio (2010) or He et al. (2015b) are not appropriate for SNNs either, because SNNs have positive thresholds that are usually much larger than individual weight values. In this work, we propose simple methods for initializing parameters and normalizing backprop errors for training deep SNNs. Even though the proposed technique does not guarantee the balance of forward activations, it is effective for addressing the exploding and vanishing gradients problems. Error normalization is not critical for training SNNs with a single hidden layer. However, we observed that training deep SNNs without normalizing backprop errors mostly failed due to exploding gradients. We describe here the method in case of fully-connected deep networks for simplicity. However, the same method is also used for training convolutional SNNs.

The weight and threshold parameters of neurons in the *l*-th layer are initialized as

(10) $$\begin{array}{l} w^{\left(l\right)}\sim U\left[-\sqrt{3/M^{\left(l\right)}},\sqrt{3/M^{\left(l\right)}}\right],\;V_{th}^{\left(l\right)}=\alpha \sqrt{3/M^{\left(l\right)}},\;\alpha >1, \end{array}$$

where *U*[−*a, a*] is the uniform distribution in the interval (−*a, a*), *M*^(*l*)^ is the number of synapses of each neuron, and α is a constant. α should be large enough to stabilize spiking neurons, but small enough to make the neurons respond to the inputs through multiple layers. In general, layers with smaller number of units need to have smaller α to generate more spikes per neuron and maintain a high enough input activity for the next layer. We used values between 3 and 10 for α and tuned them for each layer to increase the learning speed, although other choices of α will lead to similar results. The weights initialized by Equation (10) satisfy the following condition:

(11) $$\begin{array}{l} E\left[\overset{M^{\left(l\right)}}{\underset{i}{\sum }}{\left(w_{ji}^{\left(l\right)}\right)}^{2}\right]=1\;\text{or }E\left[{\left(w_{ji}^{\left(l\right)}\right)}^{2}\right]=\frac{1}{M^{\left(l\right)}}. \end{array}$$

This condition is used for backprop error normalization in the next paragraph. In addition, to ensure stability, the weight parameters are regularized by decaying them so that they do not deviate too much from Equation (11) throughout training. We will discuss the weight regularization in detail in Section 2.3.1.

The main idea of backprop error normalization is to balance the magnitude of updates in weight (and in threshold) parameters among layers. In the *l*-th layer (*N*^(*l*)^ = *M*^(*l*+1)^, *n*^(*l*)^ = *m*^(*l*+1)^), we define the error propagating back through the *i*-th active neuron as

(12) $$\begin{array}{l} \delta_{i}^{\left(l\right)}=\frac{g_{i}^{\left(l\right)}}{{ḡ}^{\left(l\right)}}\sqrt{\frac{M^{\left(l+1\right)}}{m^{\left(l+1\right)}}}\overset{n^{\left(l+1\right)}}{\underset{j}{\sum }}w_{ji}^{\left(l+1\right)}\delta_{j}^{\left(l+1\right)}, \end{array}$$

where $g_{i}^{\left(l\right)}=1/V_{th,i}^{\left(l\right)}$, ${ḡ}^{\left(l\right)}=\sqrt{E\left[{\left(g_{i}^{\left(l\right)}\right)}^{2}\right]}≅\sqrt{\frac{1}{n^{\left(l\right)}}\overset{n^{\left(l\right)}}{\underset{i}{\sum }}{\left(g_{i}^{\left(l\right)}\right)}^{2}}$. Thus, with Equation (11), the expectation of the squared sum of errors can be maintained constant through layers (i.e., $E\left[\overset{n^{\left(l\right)}}{\underset{i}{\sum }}{\left(\delta_{i}^{\left(l\right)}\right)}^{2}\right]=1$ for all layers). Although this was confirmed for the case without a WTA circuit, we found that it still approximately holds for networks using WTA. The discrepancy could easily be corrected by introducing additional parameters in Equation (12), but all results presented in this paper were obtained with the simpler version. Weight and threshold parameters are updated as:

(13) $$\begin{array}{l} \Delta w_{ij}^{\left(l\right)}=-\eta_{w}\sqrt{\frac{N^{\left(l\right)}}{m^{\left(l\right)}}}\delta_{i}^{\left(l\right)}{\hat{x}}_{j}^{\left(l\right)},\;\Delta V_{th,i}^{\left(l\right)}=-\eta_{th}\sqrt{\frac{N^{\left(l\right)}}{m^{\left(l\right)}M^{\left(l\right)}}}\delta_{i}^{\left(l\right)}{â}_{i}^{\left(l\right)}, \end{array}$$

where η_*w*_ and η_*th*_ are the learning rates for weight and threshold parameters, respectively. We found that the threshold values tend to decrease through the training epochs due to SGD decreasing the threshold values whenever the target neuron does not fully respond to the corresponding input. Small thresholds, however, could lead to exploding firing rates within the network. Thus, we used smaller learning rates for threshold updates to prevent the threshold parameters from decreasing too much. $\hat{x}$ and â in Equation (13) are the effective input and output activities defined as: ${\hat{x}}_{j}=x_{j}$, ${â}_{i}=\gamma a_{i}-\sigma \overset{n}{\underset{j\neq i}{\sum }}\kappa_{ij}a_{j}$. By using Equation (13), at the initial stage of training, the amount of updates depends on the expectation of per-synapse activity of active inputs, regardless of the number of active synapses or neurons. Thus, we can balance updates among layers in deep SNNs.

### 2.3. Regularization

As in conventional ANNs, regularization techniques such as weight decay during training are essential to improve the generalization capability of SNNs. Another problem in training SNNs is that because thresholds need to be initialized to large values as described in Equation (10), only a few neurons respond to input stimuli and many of them remain silent. This is a significant problem, especially in WTA circuits. In this section we introduce weight and threshold regularization methods to address these issues.

#### 2.3.1. Weight regularization

Weight decay regularization is used to improve the stability of SNNs as well as their generalization capability. Specifically, we want to maintain the condition in Equation (11). Conventional L2-regularization was found to be inadequate for this purpose, because it leads to an initial fast growth, followed by a continued decrease of weights. To address this issue, a new method named exponential regularization is introduced, which is inspired from max-norm regularization (Srivastava et al., 2014). The cost function of exponential regularization for neuron *i* of layer *l* is defined as:

(14) $$\begin{array}{l} L_{w}\left(l,i\right)=\frac{1}{2}\lambda e^{\beta \left(\sum_{j}^{M^{\left(l\right)}}{\left(w_{ij}^{\left(l\right)}\right)}^{2}-1\right)}, \end{array}$$

where β and λ are parameters to control the balance between error correction and regularization. Its derivative with respect to a weight parameter can be written as (for the purpose of comparison with L2 and max-norm regularization):

(15) $$\begin{array}{l} \frac{\partial L_{w}\left(l,i\right)}{\partial w_{ij}^{\left(l\right)}}=\beta \lambda \times w_{ij}^{\left(l\right)}\times e^{\beta \left(\sum_{j}^{M^{\left(l\right)}}{\left(w_{ij}^{\left(l\right)}\right)}^{2}-1\right)} \end{array}$$

L2-regularization has a constant rate of decay regardless of weight values, whereas max-norm regularization imposes an upper bound of weight increase. Exponential regularization is a compromise between the two. The decay rate is exponentially proportional to the squared sum of weights. Thus, it strongly prohibits the increase of weights like max-norm regularization. Weight parameters are always decaying in any range of values to improve the generalization capability as in L2-regularization. However, exponential regularization prevents weights from decreasing too much by reducing the decay rate. Thus, the magnitude of weights can be easily maintained at a certain level.

#### 2.3.2. Threshold regularization

Threshold regularization is used to balance the activities among *N* neurons receiving the same input stimuli. This mechanism prevents the occurrence of too many dead neurons, and thereby improves accuracy. Threshold regularization is particularly important when WTA circuits are used, since the firing of neurons is additionally suppressed by lateral inhibition. When *N*_*w*_ neurons fire after receiving an input spike, their thresholds are increased by ρ*N*. Subsequently, for all *N* neurons, the threshold is decreased by ρ*N*_*w*_. Thus, highly active neurons become less sensitive to input stimuli due to the increase of their thresholds. On the other hand, rarely active neurons can respond more easily for subsequent stimuli. Because the membrane potentials are restricted to the range [−*V*_*th*_, *V*_*th*_], neurons with smaller thresholds, because of their tight lower bound, tend to be less influenced by negative inputs. Threshold regularization actively prevents dead neurons and encourages all neurons to equally contribute to the optimization. This kind of regularization has been used for competitive learning previously (Rumelhart and Zipser, 1985; Afshar et al., 2014). We set a lower bound on thresholds to prevent spiking neurons from firing too much due to extremely small threshold values. If the threshold of a neuron is supposed to go below the lower bound, then instead of decreasing the threshold, all weight values of the neuron are increased by the same amount. Threshold regularization was done during the forward propagation in training.

### 2.4. Objective function and training procedure

Using the regularization term from Equation (14), the objective function for each training sample (using batch size = 1) is given by

(16) $$\begin{array}{l} L=\frac{1}{2}||o-y|{|}^{2}\;+\underset{l\in \text{hidden}}{\sum }\underset{i}{\sum }L_{w}\left(l,i\right) \end{array}$$

where *y* is the label vector and *o* is the output vector. Each element of *o* is defined as $o_{i}=\#{\text{spike}}_{i}/\underset{j}{\max}\left(\#{\text{spike}}_{j}\right)$, where #spike_*i*_ is the number of output spikes generated by the *i*-th neuron of the output layer. The output is normalized by the maximum value instead of the sum of all outputs. With this scheme, it is not necessary to use weight regularization for the output layer.

The training procedure can be summarized as follows: For every training sample, e.g., an image from the MNIST database, a set of events is generated. The events are propagated forward through the network using the event-driven update rule described in Equation (1) with threshold regularization. This simulation is purely event-driven, and does not use discrete time steps. Auxiliary activity values defined in Equation (4) are also calculated for training during forward propagation. Threshold regularization and auxiliary activity values are used for training only. Thus, they are not necessary if the trained network is used later for inference. After all the events from the set have finished propagating forward through the network, the events of the output layer are counted to obtain the output vector as described above Equation (16). This is used to calculate the error vector, which is normalized as $\left(o-y\right)/\sqrt{N_{\text{nze}}}$, where *N*_nze_ is the number of nonzero elements in (*o* − *y*). The error is propagated backward through the network using the chain rule to update weight and threshold parameters. Thus, the backward phase is done once for each input sample like in the conventional frame-based backprop.

## 3. Results

MNIST is a hand written digit classification dataset consisting of 60,000 training samples and 10,000 test samples (LeCun et al., 1998). MNIST nowadays is a weak benchmark for deep learning, but it is still widely used to test new concepts, and, importantly, the only dataset for which SNN results for comparison are available. For all the results in this paper, we trained on all 60 k training samples (except for the CNN case where we used only 50 k samples and reserved 10 k samples as a validation set to determine best network parameters). We only used the 10 k test set samples for evaluation of classification accuracy.

### 3.1. Permutation invariant (PI) MNIST

The permutation-invariant (PI) version of MNIST refers to the fact that the input images are randomly permuted, resulting in a loss of spatial structure and effectively random sparse input patterns. By randomly permuting the input stimuli we prohibit the use of techniques that exploit spatial correlations within inputs, such as data augmentation or convolutions to improve performance. Using the PI MNIST thus more directly measures the power of a fully-connected classifier.

**Figure 2:**
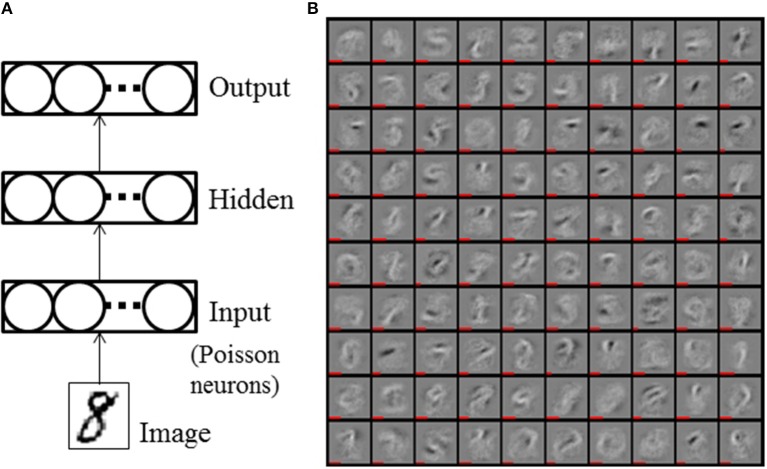
**(A)** Single hidden layer SNN. **(B)** Trained weight values of the hidden layer of 784-100-10 SNN with WTA circuit. The length of the red bars under the illustration of the weights indicates the neurons' thresholds.

Figure 2A shows the architecture of a fully connected SNN with one hidden layer (HL). An event stream is generated from a 28 × 28 pixel image of a hand written digit at the input layer, which is standard practice for SNNs (O'Connor et al., 2013; Diehl et al., 2015). The intensity of each pixel defines the event rate of Poisson events. We normalized the total event rate to be 5 keps (~43 eps per non-zero pixel on average). The accuracy of the SNN tends to improve as the integration time (i.e., the duration of the input stimuli) increases. We used a 1 second duration of the input event stream during accuracy measurements to obtain stable results. Further increase of integration time improved the accuracy only marginally (<0.1%). During training, only 50 ms presentations per digit were used to reduce the training time. In the initial phase of training deep SNNs, neuron activities tend to quickly decrease propagating into higher layers due to non-optimal weights and large thresholds. Thus, for the networks with 2 HLs, the first epoch was used as an initial training phase by increasing the duration of the input stimuli to 200 ms. Learning rate and threshold regularization were decayed by exp(−1/35) every epoch. Typical values for parameters are listed in Table 1.

**Table 1:** **Values of parameters used in the experiments**.

**Parameters**	**Values**	**Used in**
τ_*mp*_	20 ms (MNIST), 200 ms (N-MNIST)	Equations (1) and (4)
*T_ref_*	1 ms	Equation (1)
α	3−10	Equation (10)
η_*w*_	0.002−0.004	Equation (13)
η_*th*_	0.1η_*w*_ (SGD), η_*w*_ (ADAM)	Equation (13)
β	10	Equation (14)
λ	0.002−0.04	Equation (14)
ρ	0.00004−0.0002	Section 2.3.2

**Figure 3:**
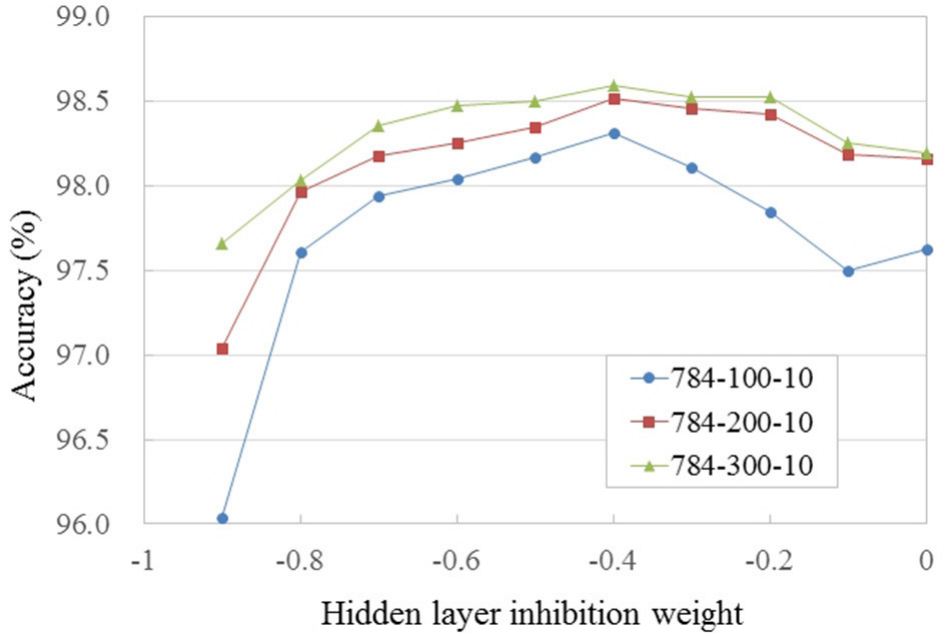
**Accuracy vs. strength of lateral inhibition in the hidden layer for PI MNIST**. Networks were trained with the same initial weights. Values are averaged over epochs [131, 150].

We trained and evaluated SNNs with differently sized hidden layers (784-*N*-10, where *N* = 100, 200, 300) and varied the strength of lateral inhibitory connections in WTA circuits (in the HL and the output layer) to find their optimum value. All the networks were initialized with the same weight values and trained for 150 epochs. The reported accuracy is the average over epochs [131, 150], which reduces the fluctuation caused by random spike timing in the input spike stream and training. Figure 2B shows the trained weight and threshold (red bar width) values of the HL of a 784-100-10 SNN with WTA circuit. It is clearly observed that the WTA circuit leads to a sparse representation. Figure 3 shows the accuracy measured by varying the lateral inhibition strength in the HL. As the figure shows, the best performance was obtained when the lateral inhibition was at −0.4, regardless of *N*. For the output layer, we found that −1.0 gave the best result. Table 2 show the accuracies of various shallow and deep architectures in comparison with previous reports. For the deep SNNs with 2 HLs, the first HL and the output layer were competing as WTA circuits. The strength of the lateral inhibition was −0.4 and −1.0 for each one as in the case of the SNNs with 1 HL. However, for the second HL, the best accuracy was obtained without a WTA circuit, which possibly means that the outputs of the first hidden layer cannot be sparsified as much as the original inputs without losing information. The strength of the lateral inhibition could be learned instead of hand-tuned, but no improvement through learning was noticed. The best accuracy (98.64%) obtained from the SNN with 1 HL matched to that of the shallow ANN (i.e., MLP) (98.4%) and the previous state-of-the-art of deep SNNs (98.64%) (Diehl et al., 2015; Hunsberger and Eliasmith, 2015). We attribute this improvement to the use of WTA circuits and the direct optimization on spike signals. The best accuracy of a SNN with 2 HLs was 98.7% with vanilla SGD. We used the ADAM (Adaptive Moment Estimation) learning method to improve the accuracy (Kingma and Ba, 2014). This method has been shown to accelerate learning in numerous deep learning experiments. It computes adaptive learning rates for each parameter based on exponentially decaying averages of past gradients and squared gradients. By applying the ADAM learning method (β_1_ = 0.9, β_2_ = 0.999, ϵ = 10^−8^), we could further improve the best accuracy up to 98.77%, which is close to ANNs trained with Dropout or DropConnect (Wan et al., 2013; Srivastava et al., 2014) as shown in Table 2. Since there is no additional information contained in the precise spike timing in the MNIST task, these results demonstrate that our presented method achieves competitive results on standard machine learning benchmarks.

**Table 2:** **Comparison of accuracy of different models on PI MNIST**.

**Network**	**# units in HLs**	**Test accuracy (%)**
ANN (Srivastava et al., 2014)	800	98.4
ANN (Srivastava et al., 2014), Drop-out	4096–4096	98.99
ANN (Wan et al., 2013), Drop-connect	800–800	98.8
ANN (Goodfellow et al., 2013), maxout	240 × 5–240 × 5	99.06
SNN (O'Connor et al., 2013)^a^^,^^b^	500–500	94.09
SNN (Hunsberger and Eliasmith, 2015)^a^	500–300	98.6
SNN (Diehl et al., 2015)	1200–1200	98.64
SNN (O'Connor and Welling, 2016)	200–200	97.8
SNN (SGD, This work)	800	[98.56, 98.64, 98.71]^*^
SNN (SGD, This work)	500–500	[98.63, 98.70, 98.76]^*^
SNN (ADAM, This work)	300–300	[98.71, 98.77, 98.88]^*^

We compare only to models that do not use unsupervised pre-training or data augmentation, with the exception of O'Connor et al. (2013) and Hunsberger and Eliasmith (2015).

a *pretraining*,

b *data augmentation*,

* [min, average, max] values over epochs [181, 200].

### 3.2. Spiking convolutional neural network on MNIST

Convolutional neural networks (CNNs) are currently the most popular architecture for visual recognition tasks. Since CNNs can effectively make use of the spatial structure of the visual world, we tested them on the standard MNIST benchmark (LeCun et al., 1998) with data augmentation, instead of the previously used permutation invariant version. In SNNs, the state of the art accuracy on MNIST has been achieved using CNNs (Diehl et al., 2015). Here we applied our method to train a spiking CNN to its best possible classification accuracy. The network has 2 stages of convolution layers, each followed by a 2 × 2 pooling layer. For the results in this paper, the pooling was configured to be effectively sum pooling by using neurons with weights of 1 and threshold of 0.8. However, it is not completely equivalent to sum pooling, because spiking neurons can be in a refractory period, and then not every input spike produces a guaranteed output spike. The convolution layers produce 20 and 50 feature maps, respectively, with kernels of size 5 × 5. The output of the second pooling layer is connected to a fully connected hidden layer with 200 neurons followed by the output layer with 10 class neurons. Elastic distortion, an effective data augmentation technique, was used on the input images to artificially increase the training set, and further improve the accuracy (Loosli et al., 2007). ADAM was used for training. Table 3 shows the test accuracy in comparison with previous results. We could achieve 99.31% accuracy with a single network. Better results for SNNs have so far only been reported using ensembles of 64 CNNs and a different preprocessing method (Esser et al., 2015). However, the results clearly show that our proposed method achieves very competitive levels of accuracy.

**Table 3:** **Comparison of accuracy of spiking CNN models on MNIST benchmark**.

**Network**	**Preprocessing**	**Ensemble**	**Test accuracy (%)**
CNN (Garbin et al., 2014)	None	1	98.3
CNN (Diehl et al., 2015)	None	1	99.1
Sparsely connected network (Esser et al., 2015)	Affine transformation	64	99.42
CNN (This work)	Elastic distortion	1	99.31

### 3.3. N-MNIST

**Figure 4:**
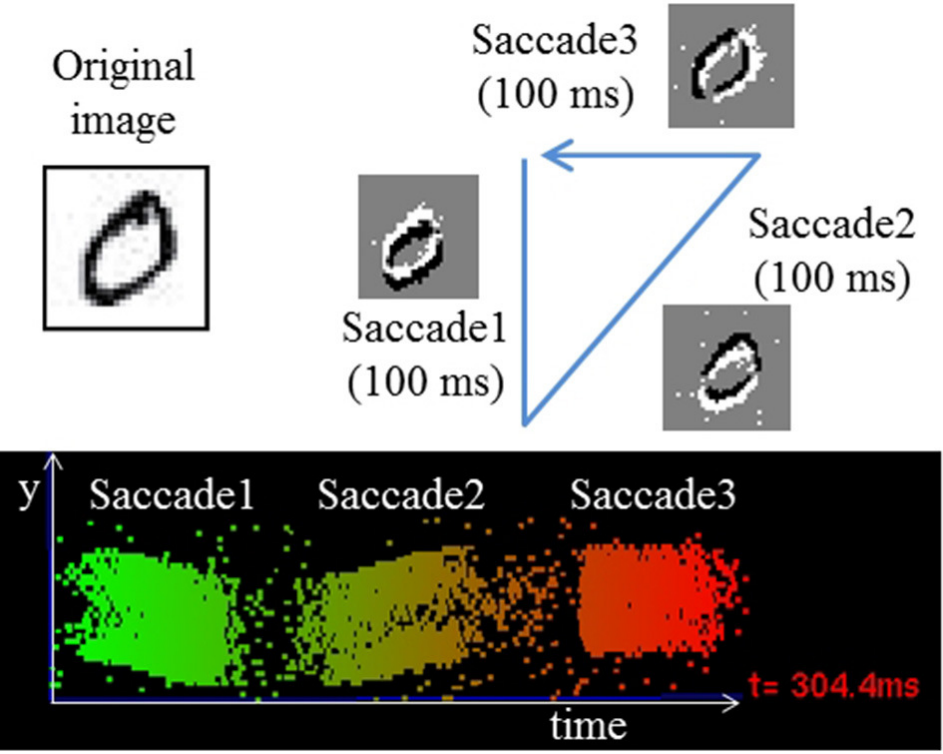
**Illustration of the saccades used to generate the N-MNIST dataset and resulting event streams (Orchard et al., 2015)**.

**Figure 5:**
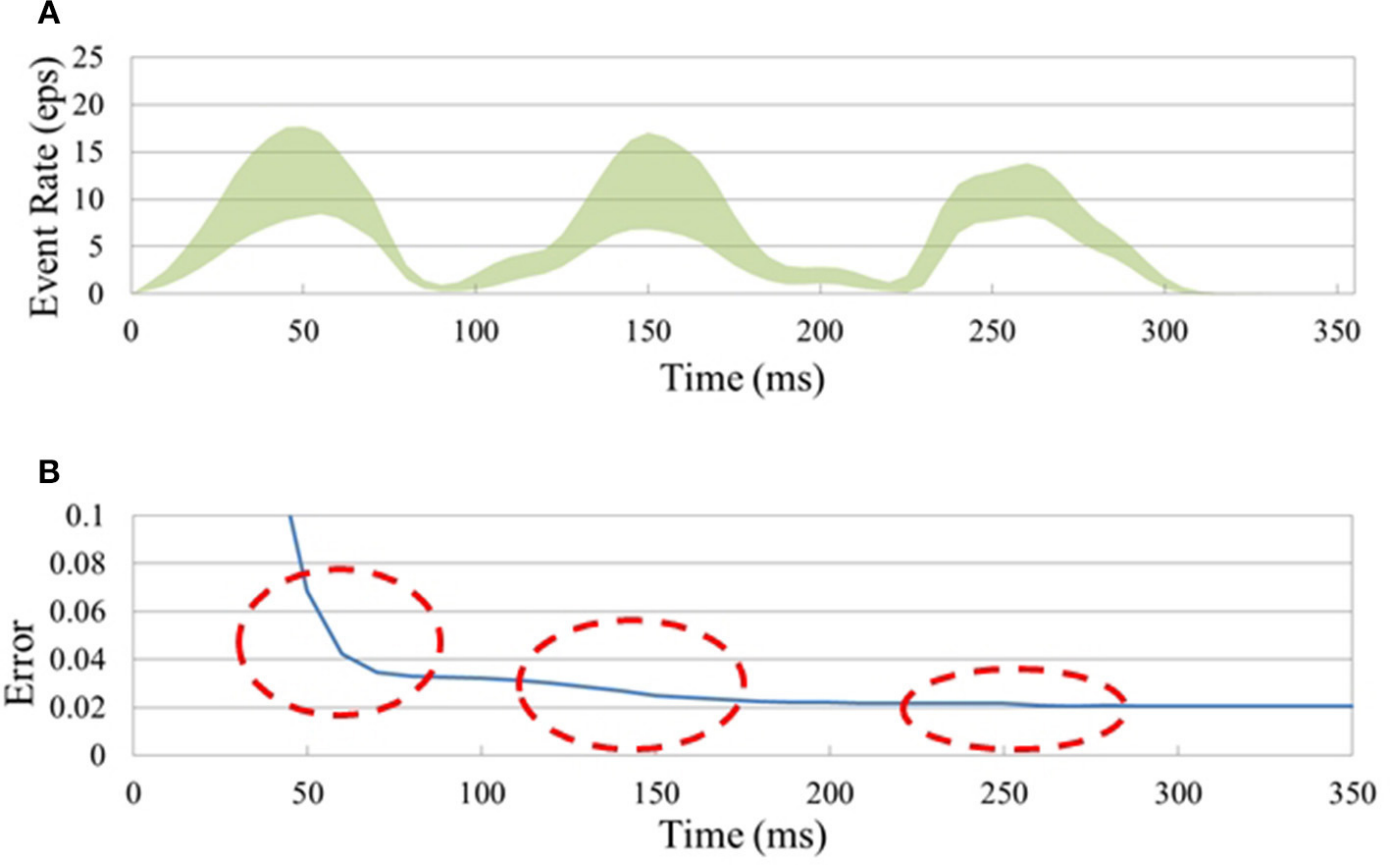
**Classification of the N-MNIST neuromorphic dataset**. **(A)** Instantaneous input event rate per pixel (i.e., total event rate divided by 34 × 34 × 2) (± std. dev.) averaged over 10,000 N-MNIST test samples. **(B)** SNNs naturally improve their accuracy over time as they integrate previous and current inference results. Big jumps in the accuracy of the (34 × 34 × 2)-200-10 SNN were observed at the times when the input event rate (see **A**) was highest (red circles).

To investigate the potential of the proposed method for training directly on event stream data, we trained a simple fully connected networks with 1 HL on the N-MNIST dataset, a neuromorphic version of MNIST (Orchard et al., 2015). As shown in Figure 4, it was generated by panning and tilting a Dynamic Vision Sensor (DVS) (Lichtsteiner et al., 2008) in front of projected images of digits. A 3-phase saccade movement of the DVS (identical for all samples) is responsible for generating events, which shifts the position of the digit in pixel space. The event stream of each digit sample has a 300 ms period with 100 ms for each saccade (Figures 4, 5A). We increased the spatial resolution of the network input to 34 × 34 to allow space for this saccadic motion. N-MNIST poses different challenges than standard computer vision datasets in several aspects: First, the goal is recognizing event-streams coming from a real silicon retina sensor. Thus, the spike trains are not Poisson event streams, which are typically used to convert still images into spike trains. Second, N-MNIST contains dynamic spatio-temporal patterns of events since the positions of digits are changing over time due to the saccade motion. Simply accumulating events over the entire 300 ms period to generate frames for training therefore does not lead to good recognition accuracy because those frames will be blurred, but accumulating over very short times means that because of the direction of motion, some edges will be emphasized and others not visible. This makes the N-MNIST benchmark as a dynamic pattern recognition task for event-based vision sensors significantly more challenging than the static MNIST task, and a better fit for the strengths of SNNs.

The previous state-of-the-art result had achieved 95.72% accuracy with a spiking CNN (Neil and Liu, 2016). Their approach was based on Diehl et al. (2015), converting an ANN to an SNN instead of directly training on spike trains. This led to a large drop of accuracy after conversion (98.3% → 95.72%), even though the event streams were pre-processed to center the position of the digits. In this work, however, we train and test directly on the original uncentered data. Thus, the SNN has to learn how to recognize dynamic spatio-temporal patterns of events rather than purely spatial patterns. For training, 300 consecutive events were picked at random time positions from each of the training digit event streams (about 8% of the average of about 4k total events per digit), whereas the full event streams were used for evaluating the test accuracy. Since the DVS generated two types of events (on-events for intensity increase, off-events for intensity decrease), we separated events into two channels based on the event type, which made the input layer size 34 × 34 × 2. Table 4 shows that our result of 98.66% accuracy, or 1.34% error rate with 800 hidden units (i.e., (34 × 34 × 2)-800-10 SNN) is the best N-MNIST result with SNNs reported to date (even better than those obtained for non-spiking CNNs). Our method improves the best previously-reported ANN result of 1.7% error rate, and in addition achieves an almost 3 times smaller error rate than the best previous spiking CNN (4.28%). It is also far better than a fully-connected SNN with 10 k hidden units (7.13%) in Cohen et al. (2016) even though our network uses only 800 hidden units. This result clearly shows the importance and possible benefits of training SNNs directly on event streams.

**Table 4:** **Comparison of accuracy of different models on N-MNIST**.

**Network**	**# units in HLs**	**Centering**	**Test accuracy (%)**
ANN (Neil and Liu, 2016)	CNN	Yes	98.3
SNN (Neil and Liu, 2016)	CNN	Yes	95.72
SNN (Cohen et al., 2016)	10,000	No	92.87
SNN (This work)	800	No	[98.56, 98.66, 98.74]^*^

* [min, average, max] values over epochs [181, 200].

An SNN continuously generates output spikes, thereby improving the accuracy as it integrates input events over time. Each output spike can be interpreted as an instantaneous inference based on a small set of input spikes over a short period preceding the spike. This is true for dynamic spatio-temporal event patterns like the N-MNIST task as shown in Figure 5 ((34 × 34 × 2)-200-10 SNN). Figure 5A shows the instantaneous input event rate per pixel (i.e., total event rate divided by 34 × 34 × 2) averaged over 10,000 N-MNIST test samples. Figure 5B shows how the classification error drops as more events are accumulated from the successive saccades; the dramatic initial drop shows that for most digits most of the information about the digit is available from the first saccade already. Each subsequent saccade approximately halves the error rate.

**Figure 6:**
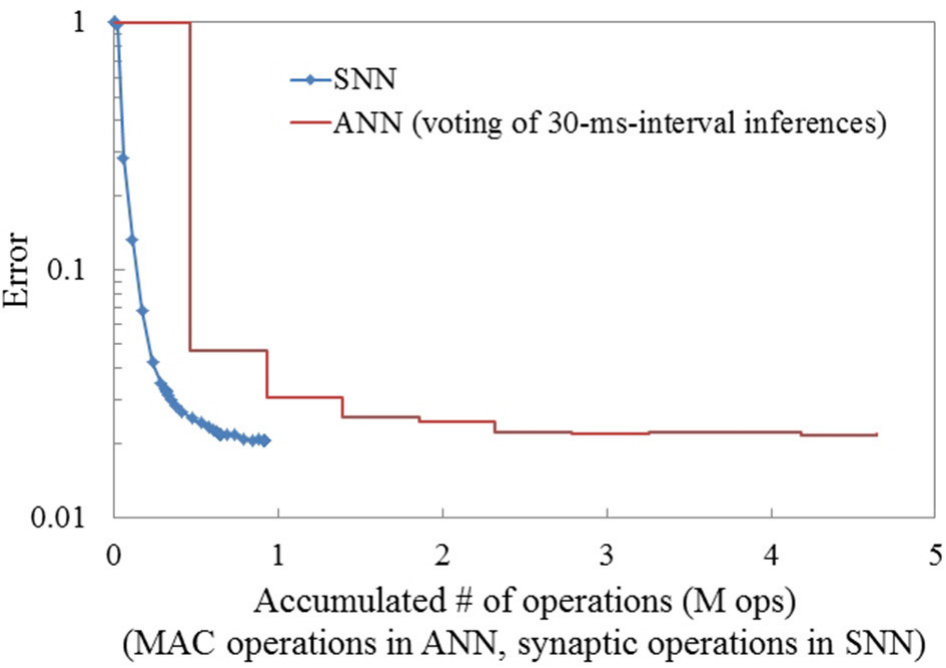
**Comparison of the computational cost (# of MAC operations for ANN, # synaptic operations for SNN) for inference between ANN and SNN in the N-MNIST task**. SNN and ANN have the same architecture: (34 × 34 × 2)-200-10. To address the movement of digits in the ANN case, the input spike streams were accumulated for 30 ms and turned into frames. Subsequently, inference for each frame was integrated over time to improve the accuracy. The SNN reaches its best accuracy long before the ANN, which requires integrating multiple frames to reach similar accuracy.

Integration of inference for dynamic pattern recognition can also be achieved in ANNs by iteratively performing inference over multiple consecutive images and using a majority vote as the predicted output. To investigate this, we trained an ANN with the same network architecture as the SNN, but using images of accumulated events over consecutive 30-ms intervals. Since we generated frames from the events over only short periods, preprocessing such as stabilizing the position of digits was not required. No significant blurring caused by saccade motion was observed in the generated frames. The test accuracy for each single snapshot image using the ANN was 95.2%. This can be interpreted as an instantaneous inference in ANNs. To obtain the final prediction, we accumulated the outputs of the softmax layer for 10 frames. When combining the results over 10 image frames (i.e., 300 ms in total), the error rate of the ANN drops to 2.2%. This accumulation of predictions reduced the gap between the ANN and SNN in term of accuracy practically to zero, however, it increased the computational cost for inference in the ANN far beyond that of the SNN. Figure 6 compares the computational cost (in terms of synaptic operations: 1 synaptic operation corresponds to 1 MAC in ANNs) for the N-MNIST task between an SNN and an ANN using accumulation across multiple frames. The computational cost for the ANN increased dramatically (around 4.8 times) compared to the SNN reaching a similar classification performance. Precise comparison of computational cost in energy is not feasible at this moment since adequate hardware for SNN is not available. Nevertheless, it clearly shows the benefit of event-driven computation in SNNs for fast and accurate inference on event-stream inputs

## 4. Discussion

We proposed a variant of the classic backpropagation algorithm, known as the most widely used supervised learning algorithm for deep neural networks, which can be applied to train deep SNNs. Unlike previously proposed techniques based on ANN-to-SNN conversion methods (Diehl et al., 2015; Esser et al., 2015; Hunsberger and Eliasmith, 2015), our method can optimize networks by using real spike events from neuromorphic vision sensors during training. We found that regularization of weight and threshold parameters are critical to stabilize the training process and achieve good accuracy. We also proposed a novel normalization technique for backpropagating error gradients to train deep SNNs. We have shown that our novel spike-based backpropagation technique for multi-layer fully-connected and convolutional SNNs works on the standard benchmarks MNIST and PI MNIST, and also on N-MNIST Orchard et al. (2015), which contains spatio-temporal structure in the events generated by a neuromorphic vision sensor. We improve the previous state-of-the-art accuracy of SNNs on both tasks and achieve accuracy levels that match those of conventional deep networks. Closing this gap makes deep SNNs attractive for tasks with highly redundant information or energy constrained applications, due to the benefits of event-based computation, and advantages of efficient neuromorphic processors (Merolla et al., 2014). We expect that the proposed technique can better capture the timing statistics of spike signals generated from event-based sensors, which is an important advantage over previous SNN training methods.

Recent advances in deep learning have demonstrated the importance of working with large datasets and extensive computational resources. The MNIST benchmark, under these considerations needs to be considered too small for evaluating the scaling of architectures and learning methods to larger applications. Furthermore, the dataset is not meant as a benchmark for SNNs, because it does not provide spike events generated from real sensors. Nevertheless, it remains important since new methods and architectures are still frequently evaluated on MNIST. In particular, almost all recently published SNN papers are tested on MNIST, where it remains the only dataset allowing comparisons. The N-MNIST benchmark (Orchard et al., 2015), which was recorded directly with neuromorphic vision sensors, is a more meaningful testbed for SNNs, even though it is still small in size, similar to the original MNIST. The fact that all events were generated following the same saccade patterns for all samples was a choice made by the creators of the dataset, and might lead to networks learning the particular spatial patterns of the saccades. It is thus unknown how classifiers trained on N-MNIST would generalize to different movement patterns, and possibly the accuracy for arbitrary saccade patterns would degrade.

Just as hardware acceleration through GPUs has been critical to advance the state of the art in conventional deep learning, there is also an increasing need for powerful hardware platforms supporting SNN training and inference. Parallelizing event-based updates of SNNs on current GPU architectures remains challenging (Nageswaran et al., 2009), although the option of simply time-stepping the simulated SNNs on GPUs has not been carefully evaluated yet. Neuromorphic processors (Camunas-Mesa et al., 2012; Merolla et al., 2014; Indiveri et al., 2015) are improving to make inference in deep networks more efficient and faster (Esser et al., 2016), but applying the training methods introduced in this paper additionally at least requires the measurement of spike statistics during runtime. The limited numerical precision of neuromorphic hardware platforms may require further adaptations of the training method, hence, at this point a hardware speed-up of onchip SNN training is not yet feasible, but remains an important topic for further research. It may be that a platform such as SpiNNaker (Furber et al., 2013), which consists of a mesh of specialized ARM processors, could be used to simulate the forward propagation through the SNN while simultaneously collecting the necessary statistics for backprop training.

Here we have presented only examples where spiking backpropagation was applied to feed-forward networks, but an attractive next goal would be to extend the described methods to recurrent neural networks (RNNs) (Schmidhuber, 2015), driven by event-based vision and audio sensors (Neil and Liu, 2016). Here the advantages of event-based sensors for sparsely representing precise timing could be combined with the computational power of RNNs for inference on dynamical signals.

## Author contributions

JL developed the theory and performed the experiments. JL, TD, and MP wrote the paper.

### Conflict of interest statement

The authors declare that the research was conducted in the absence of any commercial or financial relationships that could be construed as a potential conflict of interest.
